# Clinical practice guidelines for the management of neuropathic pain: a systematic review

**DOI:** 10.1186/s12871-015-0150-5

**Published:** 2016-02-18

**Authors:** Yunkun Deng, Lei Luo, Yuhuai Hu, Kaiyun Fang, Jin Liu

**Affiliations:** 1 Department of Anesthesiology, Guizhou Province People’s Hospital, Guiyang, Guizhou China; 2 Department of Anesthesiology, Translational Neuroscience Center, West China Hospital, Sichuan University, Chengdu, 610000 China; 3 Department of Research and Education, Guizhou Province People’s Hospital, Guiyang, Guizhou China

**Keywords:** Neuropathic pain, Clinical practice guidelines, AGREE-II

## Abstract

**Background:**

The management of neuropathic pain (NP) is challenging despite it being the recent focus of extensive research. A number of clinical practice guidelines (CPGs) for the management of NP have been published worldwide over the past 2 decades. This study aimed to assess the quality of these CPGs.

**Methods:**

We performed a systematic review of published CPGs for the management of NP. Three reviewers independently assessed the quality of the CPGs using the Appraisal of Guidelines Research and Evaluation II (AGREE-II) instrument, and recommendations of CPGs were also appraised.

**Results:**

A total of 16 CPGs were included. Thirteen CPGs were developed using an evidence-based approach, and the remaining CPGs were produced by consensus panels. None of CPGs obtained a score greater than 50 % in all six AGREE II instrument domains mainly owing to poor performance in the “Applicability” domain. The highest score of the CPGs was achieved in “Clarity and Presentation” domain, followed by “Scope and Purpose” and “Editorial Independence” domains, and the lowest scores were found the in “Applicability” domain. The majority of the CPG recommendations on the management of patients with NP were relatively consistent, especially regarding the recommendation of stepwise treatment with medication.

**Conclusions:**

Greater efforts are needed not only to improve the quality of development and presentation of the CPGs, but also to provide more efficacy evidence for the management of patients with NP.

**Electronic supplementary material:**

The online version of this article (doi:10.1186/s12871-015-0150-5) contains supplementary material, which is available to authorized users.

## Background

Clinical practice guidelines (CPGs), defined as “statements that include recommendations intended to optimize patient care that are informed by a systematic review of the evidence and an assessment of the benefits and harms of alternative care options”, have been expected to facilitate more consistent, effective and efficient medical practice, and ultimately improve health outcomes [1, 2]. Many organizations worldwide have published CPGs on similar topics in the past several decades, but their quality has been found to vary highly [3–5]. Therefore, concerns have risen about the quality of CPGs.

Neuropathic pain (NP), caused by a somatosensory lesion or various diseases, comprises a wide range of heterogenous conditions [6]. NP affects millions of people worldwide, and its estimated prevalence in the general population is as high as 7–8 % [7, 8]. Generally, NP is chronic, severe and resistant to over-the-counter analgesics. Thus, the management of NP is challenging. To improve the management for NP, the European federation of Neurological Societies (EFNS) [9–13], the Canadian Pain Society Special Interest Group on Neuropathic Pain (NePSIG) [14–16], the Assessment Committee of the Neuropathic Pain Special Interest Group of the International Association for the Study of Pain (IASP) [17–19], the National Institute for Health and Care Excellence (NICE) [20, 21], as well as an expert panel of the Middle East region (MER) [22], Latin American (LA) [23], and South Africa (SA) [24], have developed the CPG for the management of NP.

In the present study, we systematically reviewed the available CPGs for NP, focusing on their methodological quality, using the Appraisal of Guidelines Research and Evaluation II (AGREE II) instrument, and also assessed the consistency of the CPG recommendations.

## Methods

### Search strategy and CPG selection

Two experienced systematic reviewers searched relevant studies to identify CPGs for the management of NP. The following electronic databases were searched: MEDLINE, Embase, the National Guideline Clearinghouse, the Guidelines International Network, and Canadian Medical Association Infobase. Additionally, we searched the websites of the related associations, institutes, societies, and communities, including EFNS, the Canadian Pain Society, IASP, and the South African Society of Anaesthesiologists.

The following terms and Boolean operators were used in Mesh and free-text searches: (Practice Guideline OR Guideline OR Consensus OR Recommendation) and neuropathic pain. Finally, we also scanned the reference lists of relevant published articles not identified in the database searches.

### Eligibility criteria

Two reviewers independently examined and selected the CPGs according to inclusion and exclusion criteria. The inclusion criteria were as follows: (1) explicit statement identifying itself as a “guideline”; (2) CPGs that included recommendations concerning screening, diagnosis, and/or management for neuropathic pain; (3) CPGs that included a systematic review of the evidence; (4) CPGs produced by the related associations, institute, societies, or communities; (5) CPGs published in English. The exclusion criteria were as follows: (1) consensus statements, which were derived from the collective opinion of an expert panel not based on a systematic review of the evidence; (2) articles including primary studies, narrative reviews, text-like documents on development methods, documents on comments related to CPGs; (3) documents focused entirely on a unique condition, such as cancer-related neuropathic pain, postherpetic neuralgia, diabetic neuropathy, among others.

### Selection of CPGs

Following completion of all searches, references were merged and duplicates were removed. By reading titles and abstracts, two reviewers independently scanned the references to verify their eligibility using the pre-defined inclusion and exclusion criteria listed above. Additionally, two reviewers independently scanned the full-text to further verify their eligibility according to the eligibility criteria after the full-text article was obtained. If needed, disagreements were resolved by discussion with a third reviewer.

### Appraisal of selected CPGs using AGREE II instrument

The AGREE II instrument, an updated version of the original AGREE instrument, is an international, rigorously developed, and validated instrument used widely to assess CPGs [25]. It consists of 23 key items organized into six domains: “scope and purpose” (items 1–3), “stakeholder involvement” (items 4–7), “rigor of development” (items 8–14), “clarity of presentation” (items 15–18), “applicability” (items 19–21), and “editorial independence” (items 22–23). Each item in a domain is scored from 1 (strongly disagree) to 7 (strongly agree). A score of 1 (strongly disagree) should be given if there is no relevant information on the AGREE II items or this information is very poorly reported. A score of 7 (strongly agree) should be assigned when the full criteria and considerations articulated in User’s Manual have been met. A score between 2 and 6 should be given if reporting information does not meet the full criteria or considerations relevant to the AGREE II item [26]. Three reviewers assessed each included CPG independently and provided their scores on the overall assessment. Item scores were discussed and scoring discrepancies were solved by consensus. The score for each domain was calculated as follows: (obtained score-minimal possible score)/(maximal possible score-minimal possible score). As defined by AGREE II, we considered a CPG as satisfactory if it scored at least 50 % on all six domains [27].

### Statistical analysis

Descriptive and statistical analyses were performed for each domain of the AGREE II instrument. Inter-rater reliability within each domain was examined using the intra-class correlation coefficient (ICC) with a 95 % confidence interval. The degree of agreement was classified according to the scale proposed by Landis and Koch, as follows: poor (<0.00), slight (0.00–0.20), fair (0.21–0.40), moderate (0.41–0.60), substantial (0.61–0.80) and very good or almost perfect (0.81–1.00) [28]. All statistical analyses were performed using SPSS and statistical significance was considered with *P* < 0.05.

## Results

### Study selection

**Fig. 1:**
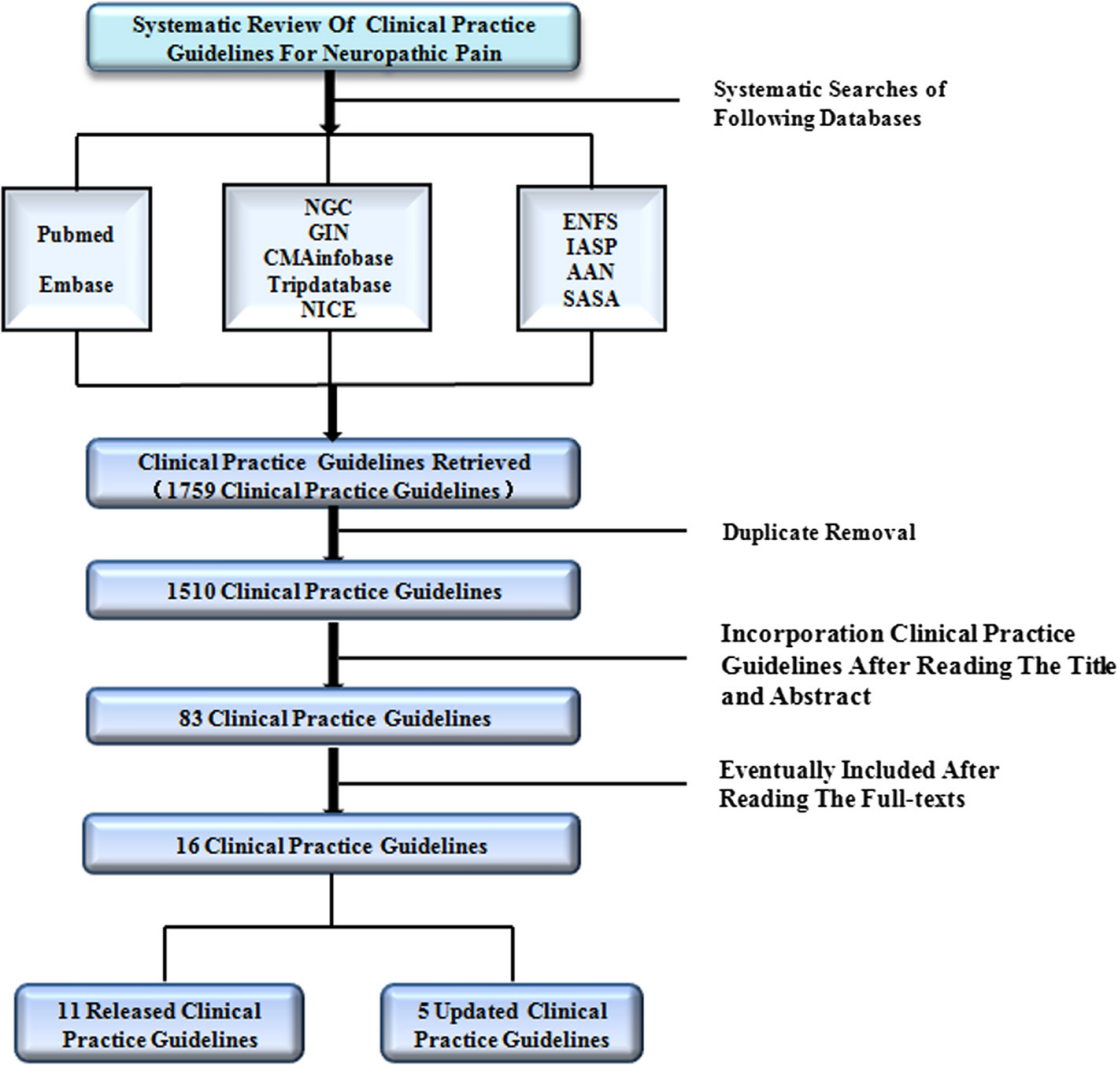
Selection process

As shown in Fig. 1, the database search identified 1759 documents. After three reviewers read the title, abstract and full-text according to the inclusion and exclusion criteria, 16 articles were eventually selected for inclusion.

### Characteristic of the CPGs

Table 1 shows the summary of the characteristics of included CPGs. The sixteen CPGs were published between 2004 and 2014. Among the 16 included CPGs, 12 CPGs were original, and five were updated versions. With the exception of the CPGs published by Latin American, South American, and Middle East regions, all the included CPGs were developed using an evidence-based approach. The 16 CPGs selected focused on the assessment and pharmacological and interventional treatments. In terms of funding, CPGs were financed independently by professional organizations, governments and academic societies, except for one CPG developed in Latin America that did not disclose a funding source. Of the sixteen selected CPGs, ten CPGs were developed by medical societies, four by governmental agencies, and two by professional organizations (Additional files 1 and 2).

**Table 1:** Summary and characteristics of NP management guidelines included in the study

Guideline name	Country or region	Release time	Developed methods	Institute	Update	Clinical problem	Funding
EFNS guidelines on neuropathic pain assessment	European	2004	Evidence-based approach	EFNS (European Federation of Neurological Societies)	1 (2009)	assessment	EFNS
EFNS guidelines on pharmacological treatment of neuropathic pain	European	2006	Evidence-based approach	EFNS	1 (2010)	pharmacological treatment	EFNS
EFNS guidelines on neurostimulation therapy for neuropathic pain	European	2007	Evidence-based approach	EFNS	0	neurostimulation therapy	EFNS
EFNS guidelines on neuropathic pain assessment:revised 2009	European	2009	Evidence-based approach	EFNS	0	assessment	EFNS
EFNS guidelines on pharmacological treatment of neuropathic pain: 2010 revision	European	2010	Evidence-based approach	EFNS	0	pharmacological treatment	EFNS
Pharmacological management of chronic neuropathic pain - Consensus statement and guidelines from the canadian pain society	Canada	2007	Evidence-based approach and Consensus statement	CPS	1 (2014)	pharmacological treatment	Pfizer Canada
Evidence-based guideline for neuropathic pain interventional treatments: spinal cord stimulation, intravenous infusions, epidural injections and nerve blocks	Canada	2012	Evidence-based approach	CPS	0	Interventional treatments: spinal cord stimulation, intravenous infusions, epidural injections and nerve blocks	CPS
Pharmacological management of chronic neuropathic pain: Revised Consensus statement and guidelines from the canadian pain society	Canada	2014	Evidence-based approach and Consensus statement	CPS		pharmacological treatment	CPS
Pharmacologic management of neuropathic pain: Evidence-based recommendations	International	2007	Evidence-based approach	NeuPSIG	0	pharmacological treatment	IASP NeuPSIG
NeuPSIG guidelines on neuropathic pain assessment	International	2011	Evidence-based approach	NeuPSIG	0	assessment	IASP NeuPSIG
Interventional management of neuropathic pain: NeuPSIG recommendations	International	2013	Evidence-based approach	NeuPSIG	0	Interventional treatments	IASP NeuPSIG
Pharmacological management of neuropathic pain in non-specialist settings: summary of NICE guidance	United Kindom	2010	Evidence-based approach	NICE	1 (2013)	pharmacological treatment	NICE
Neuropathic pain – pharmacological management: The pharmacological management of neuropathic pain in adults in non-specialist settings	United Kindom	2013	Evidence-based approach	NICE	0	pharmacological treatment	NICE
Clinical practice guidelines for management of neuropathic pain: Expert panel recommendations for South Africa	South Africa	2012	Consensus statement	painsa (Pain South Africa); NASA (Neurological Association of South Africa); PIRA (Pain Interventions and Regional Anaesthesia); SASA (South African Society of Anaesthesiologists); SASCA (South African Spinal Cord Association)	1 (2013)	neuropathic management	Pfize
Guidelines for the diagnosis and management of neuropathic pain: Consensus of a group of latin american experts	Latin America	2009	Consensus statement	FEDELAT (Latin American Federation of Chapters of the International Association for the Study of Pain)	0	neuropathic management	not mention
Guidelines for the pharmacological treatment of peripheral neuropathic pain: Expert panel recommendations for the middle east region	Middle East	2010	Consensus statement	A multidisciplinary panel of Middle East and international experts	0	pharmacological treatment	Pfizer Inc

*EFNS* european federation of neurological societies, *CPS* canadian pain society, *NeuPSIG* neuropathic pain special interest group, *NICE* national institute for health and clinical excellence

### CPGs appraisal results using the AGREE-II instrument

Three reviewers evaluated the 16 included CPGs using the AGREE II instrument. Table 2 summarizes the results of the scores for each CPG. None of the selected CPGs performed satisfactorily, that is, none achieved a score of greater than 50 % in all six AGREE II instrument domains. The highest score was obtained in the “Clarity and Presentation” domain, followed by the “Scope and Purpose”, “Editorial Independence”, “Rigor of Development”, “Stakeholder Involvement”, and “Applicability” domains. The lowest scores among all six AGREE II domains were obtained for the “Applicability” domain.

**Table 2:** Domain scores of NP management guidelines according to the AGREE II

	Domain 1	Domain 2	Domain 3	Domain 4	Domain 5	Domain 6
EFNS (2001)						
EFNS (2004)	87 %	33 %	41 %	72 %	0 %	0 %
EFNS (2006)	87 %	41 %	67 %	87 %	0 %	60 %
EFNS (2007)	87 %	41 %	61 %	91 %	0 %	60 %
EFNS (2009)	87 %	43 %	52 %	89 %	14 %	0 %
EFNS (2010)	87 %	43 %	55 %	89 %	14 %	60 %
CPS (2007)	54 %	69 %	44 %	91 %	0 %	60 %
CPS (2012)	82 %	52 %	69 %	91 %	0 %	100 %
CPS (2014)	54 %	61 %	55 %	91 %	0 %	60 %
NeuPSIG (2007)	78 %	33 %	48 %	91 %	0 %	93 %
NeuPSIG (2011)	76 %	35 %	65 %	85 %	0 %	60 %
NeuPSIG (2013)	87 %	39 %	51 %	57 %	0 %	93 %
NICE (2010)	85 %	91 %	88 %	91 %	35 %	60 %
NICE (2013)	85 %	63 %	86 %	91 %	42 %	60 %
LA (2009)	76 %	35 %	38 %	52 %	33 %	0 %
ME (2010)	74 %	26 %	27 %	81 %	0 %	100 %
SA (2012)	76 %	48 %	28 %	81 %	1 %	63 %

The results of the inter-rater reliability assessments among the three reviewers are presented in Table 3. Reliability of the assessment using the AGREE II instrument showed high scores in general. With the exception of Domain 4, classified as substantial, ICC score of the remaining domains was graded as very good according to the scale by Landis and Koch.

**Table 3:** Inter-class correlation coefficient for mean rater scores by AGREE domain

Domain	Intraclass correlation coefficient (95 % CI/average)	Cronbach’s alpha	F value	sig
Scope and purpose	0.857 (0.670–0.945)	0.96	25.3	0.000
Stakeholder involvement	0.899 (0.786–0.960)	0.96	26.3	0.000
Rigor of development	0.916 (0.680–0.974)	0.99	68.8	0.000
Clarity of presentation	0.704 (0.135–0.906)	0.97	30.3	0.000
Applicability	0.988 (0.974–0.996)	1	255.5	0.000
Editorial independence	0.977 (0.949–0.991)	0.99	137	0.000

### Recommendations of the CPGs

As shown in Table 4, we summarized the consensus treatment recommendations. The anticonvulsants pregabalin and gabapentin, low-dose TCAs, SSNRIs duloxetine and venlafaxine, and topical lidocaine showed efficacy for the management of NP and were recommended as first-line and second-line medications, respectively.

**Table 4:** Summary of recommendation of stepwise therapeutic agents

Recommendation level	Mechanism	Drug							
		IASP (2007)	CPS (2007)	Latin Amercian (2009)	NICE (2010)	MER (2010)	SA (2013)	Fench (2010)	Danish (2010)
First-line analgesics	Anti-epileptics (anticonvulsants)	Gabapentin	Pregabalin		Pregabalin	Pregabalin	Pregabalin	Pregabalin	Pregabalin
		Pregabalin	Gabapentin			Gabapentin	Gabapentin	Gabapentin	Gabapentin
	TCAs	Nortriptyline	TCAs	Nortriptyline	Amitriptyline	Nortriptyline	Low-dose amitriptyline	TCAs	TCAs
		Desipramine		Desipramine		Desipramine	Other TCA		
				Amitriptyline					
	Topical treatments	lidocaine patch 5 %		Topical lidocaine		Topical lidocaine		Topical lidocaine	Topical lidocaine
	SNRIs	Duloxetine					Duloxetine	Duloxetine	
		Venlafaxine					Venlafaxine		
	Opioid analgesics							Tramadol	
Seconds-line analgesics	SNRIs		Venlafaxine	Gabapentin		Duloxetine	Either increasing the dose of the current drug or adding a drug from a different class. For combination treatment, pregabalin with either an SNRI or amitriptyline.	Venlafaxine	Tramadol Opioids combination therapy
			Duloxetine	Pregabalin		Venlafaxine			
	Topical treatments		Topical lidocaine						
	Opioid analgesics	Morphine		Tramadol		Oxycodone		Tramadol	
		Oxycodone		Morphine		Tramadol			
		Methadone		Oxycodone					
		Levorphanol							
		Tramadol							
	TCAs							Maprotiline	
Third-line analgesics	Opioid analgesics		Tramadol				Tramadol recommended followed by strong opioids, or combination of first-line options with opioids.		
			Morphine						
			Oxycodone						
			Methadone						
			Levorphanol						
	Anti-epileptics (anticonvulsants)	carbamazepine							
		lamotrigine							
		oxcarbazepine							
	SSRIs	citalopram							
		paroxetine							
	sodium channel blocker	Mexiletine							
	NMDA receptor antagonists	Dextromethorphan							
		Memantine							
	Topical treatments	Topical capsaicin							
	SNRIs			Duloxetine					
				Venlafaxine					
Fourth-line analgesics	Cannabinoids		Cannabinoids	Cannabinoids					
	SSRIs		Citalopram						
			paroxetine						
	Anti-epileptics (anticonvulsants)		Lamotrigine	Lamotrigine					
			Clonidine	carbamazepine					
	sodium channel blocker		mexiletine						
	Synthetic opioid		Methadone						

*TCAs* tricyclic antidepressants, *SNRIs* serotonin–norepinephrine reuptake inhibitors, *SSRIs* selective serotonin reuptake inhibitors

## Discussion and conclusions

In the present study, we evaluated the quality of CPGs and consistency of the recommendations of CPGs for the management of NP to assist physicians in the selection of the appropriate recommendations. Our review demonstrated that the overall quality of the CPGs based on the AGREE II instrument was poor. However, we found consistency in the recommendations stated in the 16 CPGs with respect to drug treatment.

The AGREE II instrument allows the evaluation of various aspects of the guidelines, including integrity, reproducibility, and transparency of guidelines among six domains. Each domain has a different value and concern. Domain 1, “Scope and Purpose,” is concerned with the overall aim of the guideline, the specific health questions being addressed, and the target population (items 1–3). Domain 2, “Stakeholder Involvement,” focuses on the extent to which the guideline was developed by the appropriate stakeholders and represents the views of its intended users (items 4–6). Domain 3, “Rigor of Development,” relates to the process used to gather and synthesize the evidence, the methods to formulate the recommendations, and update them (items 7–14). Domain 4, “Clarity of Presentation,” deals with the language, structure, and format of the guideline (items 15–17). Domain 5, “Applicability” pertains to the likely barriers and facilitators to implementation, strategies to improve uptake, and resource implications of applying the guideline (items 18–21). Domain 6, “Editorial Independence” is concerned with the formulation of recommendations not being unduly biased with competing interests (items 22–23).

Our study showed that four domains concerning “Stakeholder Involvement”, “Rigor of Development”, “Applicability”, and “Editorial Independence” had serious shortcomings because the related information was poor provided. The remaining domains, including “Scope and Purpose” and “Clarity of Presentation” tended to more precisely reported because most of the included guidelines described in detail the specific and focused clinical questions they aimed to address, the target population, specific and unambiguous presentation, different management options for different presentations, and easily identifiable presentation.

Although the AGREE II instrument provides six independent domains, the “Rigor of Development” domain is considered the strongest indicator of quality of all the domains because it evaluates the integrity of the guideline development process. Among the included CPGs, low scores in the “Rigor of Development” domain were partly because of poor performance in this aspect, including the literature search and selection methods, lack of external review prior to publication, and lack of guideline updating mechanisms. Among the six domains, the quality of the “Applicability” domain also plays a significant role. A guideline developed systematically with clear recommendations based on the best available evidence could improve the healthcare practice [2]. However, in the present study, most of included guidelines did not describe facilitators and barriers to their application, did not consider the potential resource implications of applying the recommendations, and did not present monitoring or auditing criteria; thus they had a limited effect on improving healthcare quality [2, 27]. Especially for CPGs published by developing regions, treatment recommendations were restricted by the limited availability of resources faced in the respective regions. The “Applicability” domain had a great effect on the implementation of CPGs. Additionally, the “Editorial Independence” domain refers to the most frequent sources of bias in the guidelines, and it was also found to be poor among the CPGs assessed in this study. Although the authors of guidelines might have economic ties with the pharmaceutical industry or even funding from pharmaceutical companies, most guidelines fail to provide information about potential conflicts of interest.

Guidelines need to be evaluated not only for methodological quality but also for validity of their content in terms of recommendations. Thus, this study also analyzed the recommendations of these guidelines for the management of NP. In general, these recommendations were consistent on the diagnosis, assessment and pharmacological management despite scoring poorly in their rigor of development. It was difficult to tell whether we obtained these results because there was insufficient evidence to develop the guidelines or because the CPG authors did not search and make use of the best evidence available.

A large number of the drug development research has been devoted to the field of NP. A large number of analgesic agents have shown efficacy for the treatment of NP. However, no more than 40–60 % of patients have obtained sufficient pain relief with medications alone and in combination [11, 17]. Thus, it should be noted that treatment of patients with NP should be considered an integral component of a more comprehensive approach.

In general, the guidelines for the management of NP vary considerably in terms of quality, because of the apparently low standards. To reduce the variability of the CPGs, guideline development groups should have standard methodology and strategy. Further, it would be helpful to become familiarized with the AGREE II instrument domains to know what information should be reported and how it ought to be reported in the CPGs. Here, we also give some pointers to reduce the variability among the CPGs:

1) The study should describe the specific health question it aims to address, such as prevention, screening, diagnosis, or treatment, and target population, including sex, age clinical condition, severity stage of the disease, comorbidities, and others. 2) The guideline development group should include individuals from all relevant professional groups, not only or mainly health care providers. Competing interests of guideline development group members should be fully recorded and addressed. 3) In the guideline development process, appropriate methodologies and rigorous strategies are important for successful implementation of the resulting recommendations, such as the systematic methods and criteria for selecting the evidence, methods for formulating the recommendations, health benefits, side effects considered in formulating the recommendations, links between the recommendations and the evidence. Additionally, guidelines should be externally reviewed prior to their publication, and a procedure for guideline updating should be planned and described. 4) The different options for the management of the condition or health issue should be clearly presented and the recommendations should be specific and unambiguous. 5) The views of the funding body and interests of the development group should been recorded and addressed.

## Additional files

Additional file 1: **PRISMA 2009 checklist.** (DOC 63 kb)

Additional file 2: **PRISMA 2009 flow diagram.** (DOC 60 kb)

## Abbreviations

NP: Neuropathic pain
CPGs: Clinical practice guidelines
AGREE-II: Appraisal of guidelines research and evaluation II
EFNS: European federation of neurological societies
NePSIG: Canadian pain society special interest group on neuropathic pain
IASP: International association for the study of pain
NICE: National institute for health and care excellence
ICC: Intra-class correlation coefficient
MER: Middle East region
LA: Latin American
SA: South Africa
TCAs: Tricyclic antidepressants
SNRIs: Selective serotonin/noradrenaline reuptake inhibitors
