# Supplementary material for: Clinical practice guidelines for the management of neuropathic pain: a systematic review
Source: BMC Anesthesiol. 2016 Feb 18;16:12. doi: 10.1186/s12871-015-0150-5 (PMC4759966; doi:10.1186/s12871-015-0150-5)
Supplement: Supplementary file 2 — PRISMA 2009 flow diagram. (DOC 60 kb) [file 12871_2015_150_MOESM2_ESM.doc]

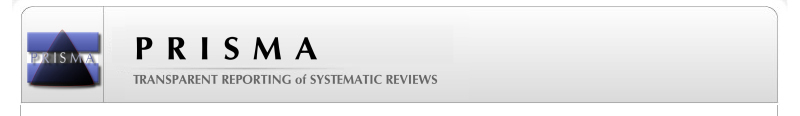
**PRISMA 2009 Flow Diagram**

**Screening**

**Included**

**Eligibility**

**Identification**

Records identified through database searching
(n = 1759 )

Additional records identified through other sources
(n =0 )

Records after duplicates removed
(n =1510 )

Records screened
(n = 83 )

Records excluded
(n =1427 )

Full-text articles assessed for eligibility
(n =16 )

Full-text articles excluded, with reasons
(n = 67 )

Studies included in qualitative synthesis
(n =16 )

Studies included in quantitative synthesis (meta-analysis)
(n =0 )
